# Chronic Thermogenic Dietary Supplement Consumption: Effects on Body Composition, Anthropometrics, and Metabolism

**DOI:** 10.3390/nu15224806

**Published:** 2023-11-17

**Authors:** Madelin R. Siedler, Christian Rodriguez, Sarah J. White, Ethan Tinoco, Brielle DeHaven, Alexandra Brojanac, Christian LaValle, Jaylynn Rasco, Lem W. Taylor, Grant M. Tinsley

**Affiliations:** 1 Energy Balance & Body Composition Laboratory, Department of Kinesiology & Sport Management, Texas Tech University, Lubbock, TX 79409, USA; msiedler@ttu.edu (M.R.S.); christian.rodriguez@ttu.edu (C.R.); sarah.white@ttu.edu (S.J.W.); ethan.tinoco@ttu.edu (E.T.); brielle.dehaven@ttu.edu (B.D.); abrojana@ttu.edu (A.B.); chlavall@ttu.edu (C.L.); jayrasco@ttu.edu (J.R.); 2 Human Performance Laboratory, School of Exercise and Sport Science, University of Mary Hardin-Baylor, Belton, TX 76513, USA; ltaylor@umhb.edu

**Keywords:** caffeine, weight loss, fat loss, body composition, thermogenic, supplement, protein, resting energy expenditure

## Abstract

Multi-ingredient thermogenic supplements can acutely increase resting energy expenditure (REE) and subjective energy. However, less is understood about the effects of chronic consumption on body composition, metabolism, and subjective variables such as mood, sleep quality, and eating behaviors. Fifty-two healthy, exercise-trained participants (50% female; mean ± SD age: 23.5 ± 3.0 years; body fat percentage: 27.3 ± 8.0%) were randomized 2:2:1 to take a whey protein supplement alone (PRO; n = 20), in combination with a thermogenic supplement (PRO + FB; *n* = 19), or no supplement at all (CON; *n* = 13) for four weeks. Body composition, anthropometric, metabolic, hemodynamic, and subjective outcomes were collected before and after the intervention. Greater changes in REE occurred in PRO + FB as compared to CON (111.2 kcal/d, 95% CI 2.4 to 219.9 kcal/d, *p* = 0.04), without significant differences between PRO and CON (42.7 kcal/d, 95% CI −65.0 to 150.3 kcal/d, *p* = 0.61) or between PRO + FB and PRO (68.5 kcal/d, 95% CI −28.3, 165.3, *p* = 0.21). No changes in hemodynamic outcomes (blood pressure and heart rate) were observed. In exercising adults, four weeks of supplementation with protein and a multi-ingredient thermogenic product maintained fasted REE as compared to no supplementation, for which a decrease in REE was observed, without differential effects on body composition, anthropometrics, or subjective variables.

## 1. Introduction

Use of dietary supplements, which are defined as orally administered products containing one or more dietary ingredients intended to supplement an individual’s diet, has grown in prevalence in the United States [1]. In fact, data from the Centers for Disease Control and Prevention indicate that over half of U.S. adults report using one or more dietary supplements over the past 30 days, with the most common products including vitamins, minerals, and omega-3 fatty acids [1]. Among athletes and active individuals in particular, additional supplements such as protein, energy drinks, and caffeine are commonly used [2]. Motivations for supplement use among athletes range from enhancing athletic performance and recovery to improving physical health and appearance [3,4].

A reduction in weight and/or fat mass is a frequently cited goal among active and athletic individuals, whether in pursuit of performance-related (e.g., increased efficiency), strategic (e.g., eligibility to compete in a certain weight class), or aesthetic goals (e.g., competing in aesthetically based sports such as bodybuilding) [5,6]. As such, in addition to manipulating energy balance via decreased intake and increased expenditure, athletes may consider using supplements that claim to promote fat loss or provide other benefits during a weight reduction phase, such as increased feelings of energy or appetite reduction. Such commonly used thermogenic supplements or “fat burners” often contain a combination of multiple compounds, including caffeine, B-vitamins, and a variety of herbal substances that claim to promote increased energy expenditure and/or fat loss. Intake of these supplements is prevalent, with approximately 34% of male and female athletes reporting use of energy drinks and 29% reporting the use of caffeine. Meanwhile, 27% of athletes report supplementing with protein [2]. Together, these data suggest that co-supplementation with multiple products promoting similar desired effects, such as improved body composition and athletic performance, may be present.

Caffeine is a naturally occurring alkaloid compound that is commonly found in coffee, tea, and cocoa [7]. There are several proposed mechanisms of action for the noted stimulating effects of caffeine, including stimulation of the central nervous system and mobilization of the intracellular calcium ion stores required for muscular contraction. Caffeine’s centrally stimulating effects are likely a result of its antagonistic action on adenosine receptors, which results in a blockade of the depressant effects of adenosine [7]. Consumption of caffeine and caffeine-containing supplements has been shown to acutely increase resting energy expenditure (REE) [8,9,10,11] as well as potentially suppress energy intake and appetite [12]. These properties, in particular, suggest a potential utility of caffeinated supplements during a deliberate period of weight or fat loss.

Additional compounds commonly found in thermogenic formulations include “fat-burning” substances purported to promote weight loss. However, the mechanisms of these supplements—from increasing fat oxidation and impairing fat absorption to suppressing appetite and increasing energy expenditure—are less well studied and understood than those of caffeine. Such compounds include L-carnitine, conjugated linoleic acid (CLA), and traditional herbal supplements and plant extracts such as *Garcinia cambogia*. L-carnitine, a substance found naturally in high concentrations in meat products, plays a critical role in the transportation of long-chain fatty acids across the inner mitochondrial membrane for oxidation [13]. Meanwhile, CLA has been suggested to promote modest weight loss by increasing lipolysis and glucose tolerance [13,14]. Supplementation with *Garcinia cambogia* and/or its active ingredient, hydroxycitric acid, has demonstrated a small effect on weight loss in some but not all studies [15]. Indeed, across investigations of these compounds, the findings are generally inconclusive. Importantly, the number and variety of products marketed as fat burners continues to exceed the pace of research on their effectiveness [13], while published randomized controlled trials in the field have notable issues related to allocation concealment, blinding of participants and personnel, attrition bias, and selective outcome reporting [15].

B-vitamins—water-soluble compounds naturally found in foods such as grain and seafood—play important roles in critical enzymatic reactions and metabolic pathways required to generate adenosine triphosphate (ATP) [16]. As all muscular contraction—including that sustained during exercise—requires ATP, this attribute makes B-vitamins a common component of ergogenic supplements such as energy drinks [17] and multi-ingredient pre-workout supplements (MIPS) [18]. However, while previous research has suggested that B-vitamins may improve exercise performance in the context of deficiency, further benefits in nutritionally replete populations have not been clearly demonstrated [17]. Furthermore, some research suggests a potential influence of B-vitamin consumption on weight management in animal models [19], though the application of these findings to humans remains to be studied. 

The acute effects of caffeine and caffeinated multi-ingredient thermogenic supplements on variables such as REE, fat oxidation, and energy and mood-state in athletic individuals is well-documented [8,9,20,21,22,23,24]. However, the actual effectiveness of these multi-ingredient supplements in promoting the desired changes in body weight and composition over time is unclear, as are the risks of undesirable effects such as changes in blood pressure, heart rate, and sleep quality. In order for athletes and active individuals to make informed choices regarding the use of thermogenic supplementation, more research examining the chronic effects of these multi-ingredient supplements on a range of important outcomes is required. Additionally, though it has been demonstrated that increased protein ingestion can help promote the maintenance of lean mass and potentially improve subjective variables such as hunger during deliberate periods of weight loss [25,26,27], the effect of protein supplementation in combination with a thermogenic supplement remains to be elucidated. Therefore, the objective of the present investigation was to examine the influence of four weeks of whey protein supplementation with or without the use of a commercially available thermogenic supplement on body composition, anthropometric, metabolic, hemodynamic, and subjective variables over time in generally healthy, trained subjects.

## 2. Materials and Methods

### 2.1. Participants

Generally healthy, non-smoking, exercise-trained adults between 18 and 40 years of age who had maintained a relatively stable body weight (i.e., no more than a gain or loss of approximately 4.5 kg based on self-report [28]) for the past three months at the time of enrollment were recruited to participate. To be eligible, participants needed to report performing any type of exercise (endurance, resistance, or concurrent training) at least two times per week for the previous six months prior to enrollment and to report consuming no less than approximately 40 mg and no more than approximately 200 mg of caffeine daily, on average, for at least four weeks prior to enrollment. Additionally, for those taking prescription medication, no changes in medication in the past month and no limitations on consumption of caffeine or other substances in the dietary supplements were required. 

### 2.2. Ethical Approval

This study was conducted according to the guidelines established by the Declaration of Helsinki and all procedures involving human subjects were approved by the Texas Tech University Institutional Review Board (IRB2022-993). Written informed consent was obtained from all subjects. Plans for data collection were also registered prospectively at clinicaltrials.gov (Clinical Trials.gov identifier: NCT05619809).

### 2.3. Pre-Assessment Standardization

Data collection consisted of an initial visit to collect baseline measures and a follow-up visit four weeks later for post-intervention assessments [29,30]. In line with best practice suggestions for the standardized assessment of body composition and REE participants were instructed to abstain from exercise and vigorous physical activity for 24 h and to abstain from all food, fluid, caffeine, alcohol, nicotine, or other substances (including both over-the-counter and prescription medications) for eight hours prior to each scheduled visit. To support adequate hydration during the visit, participants were also instructed to ingest one liter of water between their last meal and the beginning of the eight-hour abstention from fluid on the night prior to their study visit. To further standardize measurements, participants wore skin-tight clothing (e.g., compression shorts, sports bra for females) for the duration of the body composition and anthropometric assessments. 

Upon arrival at the laboratory during the initial visit, participants were screened to confirm eligibility, the investigation was verbally explained to participants, and written informed consent was obtained. At each visit, participants’ adherence to the pre-testing guidelines was confirmed. 

### 2.4. Body Composition and Anthropometric Assessments

All devices were calibrated and used in accordance with manufacturer instructions and using methods previously described in detail elsewhere [29]. After voiding their bladder, participants were instructed to remove their shoes, socks, any additional clothing, and all metal jewelry and other accessories before proceeding. Height was then measured to the nearest 0.1 cm using a stadiometer (HM200P, Charder Medical, Taichung City, Taiwan). After participants were provided with a swim cap to collect and cover all hair, body mass was assessed with a calibrated scale (Model BWB-627-A, modified Tanita Corp., Tokyo, Japan) and investigators confirmed that participant weight was between 50 and 100 kg, an eligibility criterion, before proceeding.

Next, a dual-energy X-ray absorptiometry (DXA; iDXA, General Electric, Boston, MA, USA with enCORE software version 16.10.151, 16 [SP 1]) scan was conducted to provide an estimate of baseline body fat percentage for eligibility purposes and to provide total and segmental body composition values. With the participant lying supine, a set of foam blocks and straps were used to position the participant’s feet perpendicular to the table and their hands in neutral position to each side of their body. When participants were too broad to fit within the designated scan zone of the DXA device, a reflection scan was used to estimate excluded limbs. Previous research has shown the use of reflection scans to introduce minimal error [29,31]. In addition, research staff manually adjusted region of interest lines within the enCORE software to delineate each body segment (i.e., head, trunk, and limbs). Investigators confirmed that initial body fat percentage as assessed via DXA was ≥10% for males and ≥15% for females, the final inclusion criterion, before proceeding with additional assessments. 

Anthropometric values were collected with a three-dimensional optical scanner (SS20, Size Stream, scanner version 6.3, software version 5.2.7 for Size Stream Studio). Additionally, the thickness of the abdominal subcutaneous fat layer was assessed via ultrasound (HD3, Clarius, Vancouver, BC, Canada, scanner software version 10.1.1-468+1d2507c276). The application’s Brazilian Butt Lift (BBL) setting, which has a depth of 5 cm, was generally used unless it was determined that a greater depth would be required to assess the entire subcutaneous fat layer. In this case, the Lung setting, which has a depth of 6 cm, was used. The outer edge of the probe was placed approximately 1 cm to the right of the umbilicus and perpendicular to the participant’s torso in order to standardize the images captured. Participants were instructed to relax their arms to their sides and fully exhale before an ultrasound image was captured. The distance from the outer edge of the rectus abdominus to the outer edge of the skin was measured in ImageJ (version 1.53t, National Institutes of Health) by the same assessor with the average of three images used for each timepoint.

### 2.5. Psychometric Questionnaires and Dietary Intake Assessment

Validated questionnaires were used to elicit data related to participants’ physical activity, mood, sleep habits, and eating behavior at both timepoints. These questionnaires included the International Physical Activity Questionnaire (IPAQ) short form [32], the Mood and Feelings Questionnaire (MFQ) adult self-report 33-item-long version [33], the Pittsburgh Sleep Quality Index (PSQI) [34], and the Three-Factor Eating Questionnaire (TFEQ) Revised 18-item form [35]. 

The MFQ assesses mood disturbance over the past two weeks, with a maximum score of 66 and higher scores indicating greater severity. Scores ≥ 27 are considered indicative of depression [33]. The PSQI is a validated instrument that assesses an individual’s sleep quality based on a number of different criteria, such as sleep latency, sleep duration, and the use of sleep medications [34]. Possible scores range from 0 to 21, with higher scores indicating greater sleep disturbance. The TFEQ-R18 is a revised form of the Three-Factor Eating Questionnaire [36] that assesses eating behavior across three domains: cognitive restraint, uncontrolled eating, and emotional eating, with higher scores representing greater presence of these constructs. Maximum scores for each domain are 24, 36, and 12, respectively [35].

Female participants were additionally asked about the status and typical duration of their menstrual cycle as well as their current use of hormonal contraceptive medication. All questionnaires were administered via paper with the exception of the IPAQ, which was administered using an electronic spreadsheet (Excel, Microsoft, Redmond, WA, USA) on a laboratory computer. Lastly, participants reported all food and drink (excluding water) consumed during the full calendar day prior to the visit using the electronic Automated Self-Administered 24-Hour Dietary Assessment Tool (ASA24, National Institutes of Health, Bethesda, MD, USA). All questionnaires and reports were self-administered, though participants were provided verbal instructions necessary for completing each questionnaire and were encouraged to ask questions and request assistance from the attending investigator as needed. When administering the IPAQ, participants were instructed to report their current habitual levels of physical activity rather than their activity over the past week.

### 2.6. Resting Metabolic Rate 

Resting Metabolic Rate (RMR) was assessed via indirect calorimetry using a metabolic cart (TrueOne 2400, Parvo Medics, Salt Lake City, UT, USA) with gas and flow calibration performed each day in accordance with manufacturer instructions. The testing protocol followed current best practice guidelines for the assessment of RMR [30]. Specifically, participants were instructed to lay supine for 30 min prior to the beginning of testing and were offered a blanket for warmth if desired. Testing occurred in a quiet, dimly lit, and thermoneutral environment with an ambient temperature between 22 °C and 25 °C. Before each test began, an automated cuff (BP 785, Omron, Kyoto, Japan) was used to collect resting heart rate and blood pressure values. Then, a ventilated hood was placed over the participant’s head and expired gas collection began. The test continued until a consecutive five-minute period occurred in which the coefficient of variation (CV) was <10% for VO_2_ and VCO_2_ and <5% for RMR, with the first five minutes discarded.

### 2.7. Participant Allocation

After baseline testing was complete, participants were stratified by sex (male or female), training status (resistance-trained, endurance-trained, or concurrently trained), and body fat percentage (greater or less than 20% for males, and greater or less than 25% for females) and were allocated to an intervention using a randomly generated sequence unique to each of the 12 possible permutations of the stratification approach. The sequences were developed using the “sample” randomization function within the R software package. Participants were either allocated to the control condition (CON) or to one of two intervention groups—protein and fat burner supplementation (PRO + FB) or protein alongside a placebo supplement powder (PRO)—at a 1:2:2 ratio, respectively. The placebo was designed to look identical to the active supplement and was provided to the participants in unlabeled containers marked A or B. All participants and laboratory personnel were blinded as to which canisters contained the placebo versus the active supplement. The sole investigator who was not blinded to the identity of the supplements (GMT) did not take part in participant recruitment, communication, or data collection at any time during the study.

### 2.8. Intervention

Participants allocated to CON were instructed to limit their caffeine intake to no more than approximately 100 mg per day and to refrain from the use of any performance-enhancing or thermogenic supplement for the duration of the four-week study. If participants were already using supplements for general health support (e.g., multivitamins, greens powders, creatine) they were told to continue these as before. Otherwise, participants in CON were instructed to maintain their habitual exercise and dietary patterns, including the types of foods consumed and the frequency of meals.

Participants in PRO and PRO + FB were given the same instructions as CON but were also provided with additional supplements to consume for the duration of the four-week intervention. Both groups were provided with whey protein powder (OxyWhey Lean Wellness Protein, EHP Labs, Sydney, Australia) and were instructed to consume between one and three scoops per day based on measured body mass (1 scoop per day for participants between 50 and 59.9 kg, 2 scoops per day for participants between 60 and 89.9 kg, and 3 scoops per day for participants between 90 and 100 kg). Each 33 g scoop of the whey supplement contained approximately 130 kcal, 1.5 g fat, 2 g carbohydrate, and 25 g protein in addition to a variety of minerals, B-vitamins, and Vitamin C. Participants were instructed to consume the whey powder at any time of day and in any manner that was most conducive to daily consumption. 

In addition to the protein, participants were provided with either a powdered thermogenic supplement (OxyShred Thermogenic Fat Burner, EHP Labs, Sydney, Australia) or a visually identical placebo. As per instructions provided for the EHP Labs product, participants were instructed to begin taking one scoop of the supplement per day, either in the morning upon waking or 15 min before exercise. After one week, the participants were instructed to begin taking two scoops per day: one in the morning upon waking and the second in the early afternoon and/or 15 min before exercise. Each 4.5 g scoop of the commercially available thermogenic supplement consumed by PRO + FB contained 1 g carbohydrate and approximately 150 mg caffeine in addition to a number of vitamins, minerals, and other supplements with potentially lipolytic, mood-enhancing, and immunity-supporting properties. The full list of ingredients contained in the active supplement can be found in Table 1. The placebo powder consumed by participants in PRO contained the same flavoring ingredients as the thermogenic powder and a base of Arabic gum, which is estimated to contain 4 kcals/g (a maximum difference of 15 kcals per serving compared to the active supplement) [37].

To encourage and monitor participant compliance throughout the four-week intervention, participants were asked to complete a brief daily online questionnaire (SurveyMonkey, Inc., San Mateo, CA, USA) to elicit data pertaining to their compliance with instructed protocols the day prior. Specifically, each day, all participants were asked whether they followed their normal exercise and nutrition habits as instructed. Participants in PRO and PRO + FB were also asked if they had consumed their assigned supplement as instructed. Four weeks after their baseline assessment, each participant returned to the laboratory to complete follow-up assessments for all body composition, anthropometry, RMR, and psychometric and subjective variables. In addition, to assess the efficacy of masking, participants in PRO and PRO + FB were asked to guess the condition to which they had been assigned. At the second visit, participants in PRO and PRO + FB were asked to report their daily habitual caffeine intake only from sources outside of the provided supplement. Therefore, after the blind was removed, 300 mg caffeine (the instructed intake for weeks two through four of the intervention) were added to the values reported at the second visit in the PRO + FB group only.

### 2.9. Statistical Analysis

In the primary analysis, changes in outcome variables were evaluated using two-way mixed ANOVA with repeated measures, with group specified as a between-subjects factor and time specified as a within-subjects factor. Normality was evaluated by inspection of quantile–quantile plots, data were checked for extreme outliers (i.e., values above Q3 + 3 × IQR or below Q1–3 × IQR) using boxplot methods, and the Greenhouse–Geisser correction was applied when sphericity was violated. Additionally, homogeneity of variance was examined using Levene’s test, and homogeneity of covariances was tested using the Box’s M-test. Most variables were approximately normally distributed, contained no extreme outliers, and met assumptions of homogeneity of variance and covariance. Exceptions, which included FM, trunk FM, arm FM, RQ, WC, and select nutrient and physical activity variables, were examined both by analyzing raw data and by transforming raw data into ranks and performing two-way ANOVA with repeated measures on rank values. In these cases, there were no material differences between analyses, except for kcal intake. Therefore, to aid interpretability, the raw data were retained and are presented, with *p*-values based on ranks for kcal intake. However, variables arising from TFEQ, PSQI, and MFQ were analyzed using rank-based tests due to the ordinal nature of the outcome data. To accompany each ANOVA test, generalized eta-squared (GES) effect sizes were calculated. When a significant effect was observed, post hoc tests were performed using one-way ANOVA and pairwise *t*-tests as appropriate, with the Bonferroni correction for multiple comparisons. One participant was missing baseline REE and RQ values, which were replaced using multiple imputation with 20 iterations via the “mice” software package. To examine the potential influence of sex on changes over time, the analysis described above was supplemented by three-way ANOVA tests including sex as an additional between-subjects factor. These results are presented in the supplementary materials.

Additional analysis of change values for outcome variables was performed using one-way ANOVA. Normality and outliers were examined as previously described, and homogeneity of variance was examined using Levene’s test. Most variables were approximately normally distributed, contained no extreme outliers, and met the assumption of homogeneity of variance. Exceptions, which included changes in FM, arm FM, VAT change, RQ change, and changes in select nutrient or physical activity variables, were examined both by analyzing raw data and by using the non-parametric Kruskal–Wallis rank sum test. In these cases, there were no material differences between analyses, except for the change in protein intake. Therefore, to aid interpretability, the raw data were retained and are presented, with Kruskal–Wallis output presented for changes in protein intake. To accompany the ANOVA test, GES effect sizes were calculated. However, variables arising from TFEQ, PSQI, and MFQ were analyzed using the Kruskal–Wallis rank sum test due to the ordinal nature of the data and were accompanied by eta-squared effect sizes based on the H-statistic. When a significant effect of group was observed, Tukey’s HSD post hoc test was used. Statistical significance was accepted at *p* < 0.05, with *p*-values between 0.05 and 0.1 considered trends. Data are presented as mean ± SD unless otherwise noted. Analyses were performed using R software v. 4.3.1 [38] and the “rstatix” package v. 0.7.2 [39]. 

#### Sample Size Determination

A priori, a sample size of 50 was selected, with a goal of 20 per treatment group (PRO, PRO + FB) and 10 in the CON group. This sample size was informed by a power analysis using the partial eta-squared effect size for the BFP group × time interaction reported in a previous trial examining the effects of chronic consumption of placebo, non-caffeinated, and caffeinated dietary supplements [40]. Due to possible attrition, additional individuals were recruited such that 52 participants (PRO *n* = 20, PRO + FB *n* = 19, CON *n* = 13) completed the entire study and were included in the present analysis.

## 3. Results

### 3.1. Participant Characteristics

Of the 52 total participants (26 males and 26 females), 28 (54%) reported being concurrently trained (i.e., engaging regularly in both endurance, resistance, and/or concurrent training sessions); 20 (38%) were exclusively resistance-trained; and 4 (8%) were exclusively endurance-trained. Across the entire sample, 37 participants (71%) identified primarily as White/Caucasian, 9 (17%) identified as Hispanic/Latino, 5 (10%) identified as Asian, and 1 (2%) identified as Black/African American. Overall, participants reported having consistently trained for the past 7.9 ± 4.9 years and reported consuming approximately 144 ± 53 mg caffeine per day at the time of enrollment. At the time of enrollment, 21 (81%) of the 26 female participants reported having a regular menstrual cycle, defined as menstrual periods occurring at regular intervals and no missed periods within the past six months. A total of 12 (54%) of the 26 female participants reported using hormonal contraception. Of these, seven (58%) reported using a combined oral contraceptive pill, three (25%) used a progestin-only pill, one (8%) used an implant, and one (8%) used a hormonal intrauterine device. Baseline participant characteristics in each of the three conditions are presented in Table 2. Baseline characteristics stratified by sex and group can be found in Supplementary Table S1, and three-way ANOVA results with sex as a factor are presented in Supplementary Table S2. No meaningful three-way interactions were observed for any variable over time.

### 3.2. Participant Compliance

Compliance with completion of daily online surveys was 96.8 ± 7.2%, 91.9 ± 11.0%, and 93.5 ± 13.4% in CON, PRO, and PRO + FB, respectively. From the completed surveys, PRO participants reported following their normal nutritional habits 99.4% of the time, following their normal exercise habits 98.4% of the time, consuming their assigned supplement treatment (i.e., placebo powder) 99.6% of the time, and consuming the assigned protein supplement 98.3% of the time. PRO + FB participants reported following their normal nutritional habits 98.4% of the time, following their normal exercise habits 98.8% of the time, consuming their assigned supplement treatment (i.e., FB powder) 98.6% of the time, and consuming the assigned protein supplement 97.0% of the time. CON participants reported following their normal nutrition habits 99.2% of the time and following their normal exercise habits 97.8% of the time. 

### 3.3. Energy Intake, Expenditure, and Caffeine Consumption

Across the entire sample, at baseline, participants reported consuming 2191 ± 870 kcal, 120 ± 62 g protein, 90 ± 48 g fat, and 227 ± 86 g carbohydrate per day as assessed via the ASA24. At the second visit, participants reported consuming 1997 ± 741 kcal, 128 ± 57 g protein, 81 ± 39 g fat, and 192 ± 84 g carbohydrate. Via the IPAQ, participants reported expending 4148 ± 2483 MET-minutes per week, translating to an estimated physical activity energy expenditure of approximately 808 ± 569 kcal per day, at baseline. At the second visit, participants reported expending 3898 ± 1877 MET-minutes per week, or an estimated 744 ± 408 kcal per day. There were no significant group × time interactions observed beyond the change in reported caffeine intake, including the estimated 300 mg additional caffeine consumed by PRO + FB (Table 3). Post hoc tests for the group × time interaction for caffeine indicated that there were no differences at baseline between groups (*p* 0.36 to 0.72), but differences were present during the intervention between CON (86 mg/day) and PRO + FB (348 mg/day; *p* < 0.001) and between PRO (74 mg/day) and PRO + FB (*p* < 0.001), without a difference between CON and PRO (*p* = 1.0). Total daily carbohydrate intake decreased over time across all participants by 35 g/day, though no significant group × time interaction was present. A significant difference between groups for changes in protein intake was observed, with post hoc tests indicating a trend for difference between CON and PRO + FB (mean difference: 35 g/day; *p* = 0.08), without differences between CON and PRO (29 g/day; *p* = 0.17) or PRO and PRO + FB (6 g/day; *p* = 0.89).

### 3.4. Body Composition

**Figure 1:**
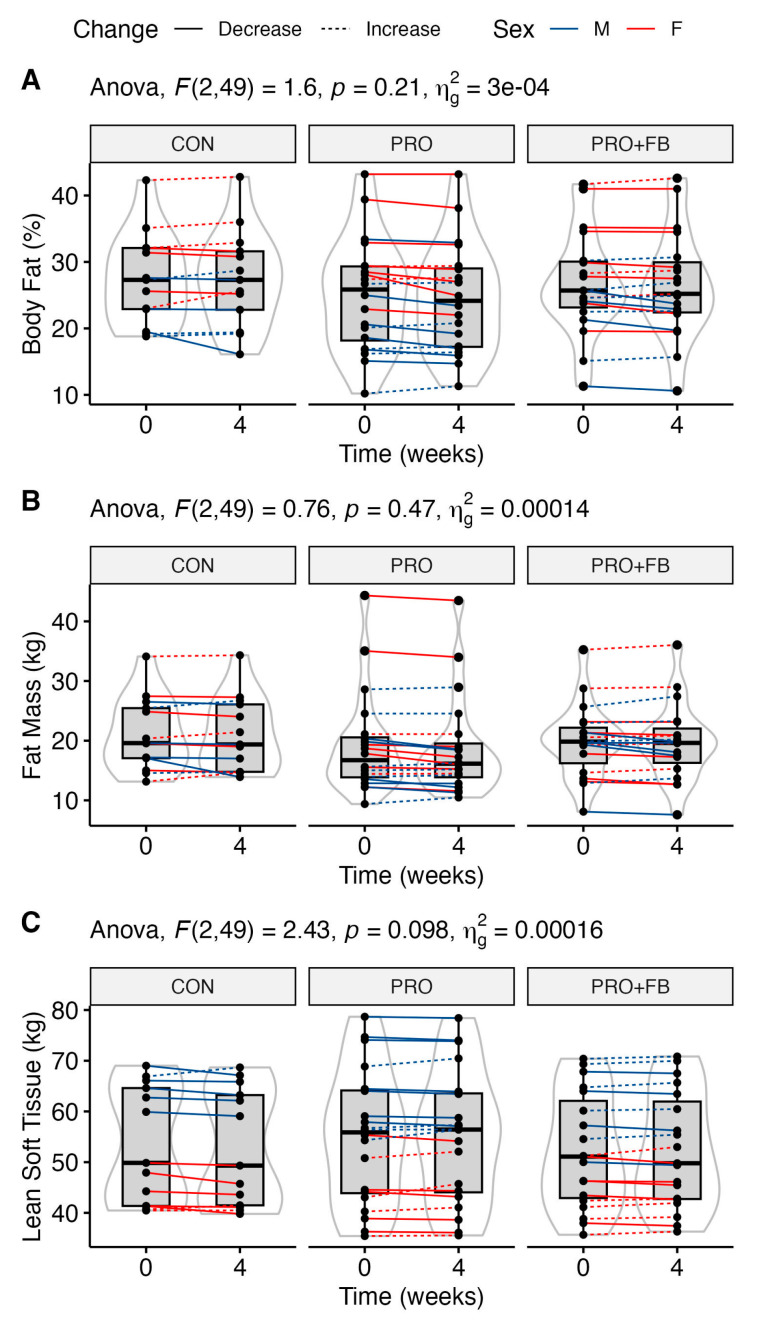
Changes in selected body composition values over the four-week intervention in each group. (**A**) Body fat percentage; (**B**) fat mass in kg; (**C**) lean soft tissue in kg.

No significant group, time, or group × time effects were observed for body composition variables (Table 3), although trends were present for time main effects for FM (*p* = 0.08) and FM_LEGS_ (*p* = 0.06), and a trend for a group × time effect for LST was observed (*p* = 0.098). Similarly, no significant differences between conditions were observed when examining change values in each condition (Table 4). Raw values and individual responses for each outcome are presented in Figure 1 and Supplementary Figures S1–S4. 

### 3.5. Body Mass and Anthropometrics

**Figure 2:**
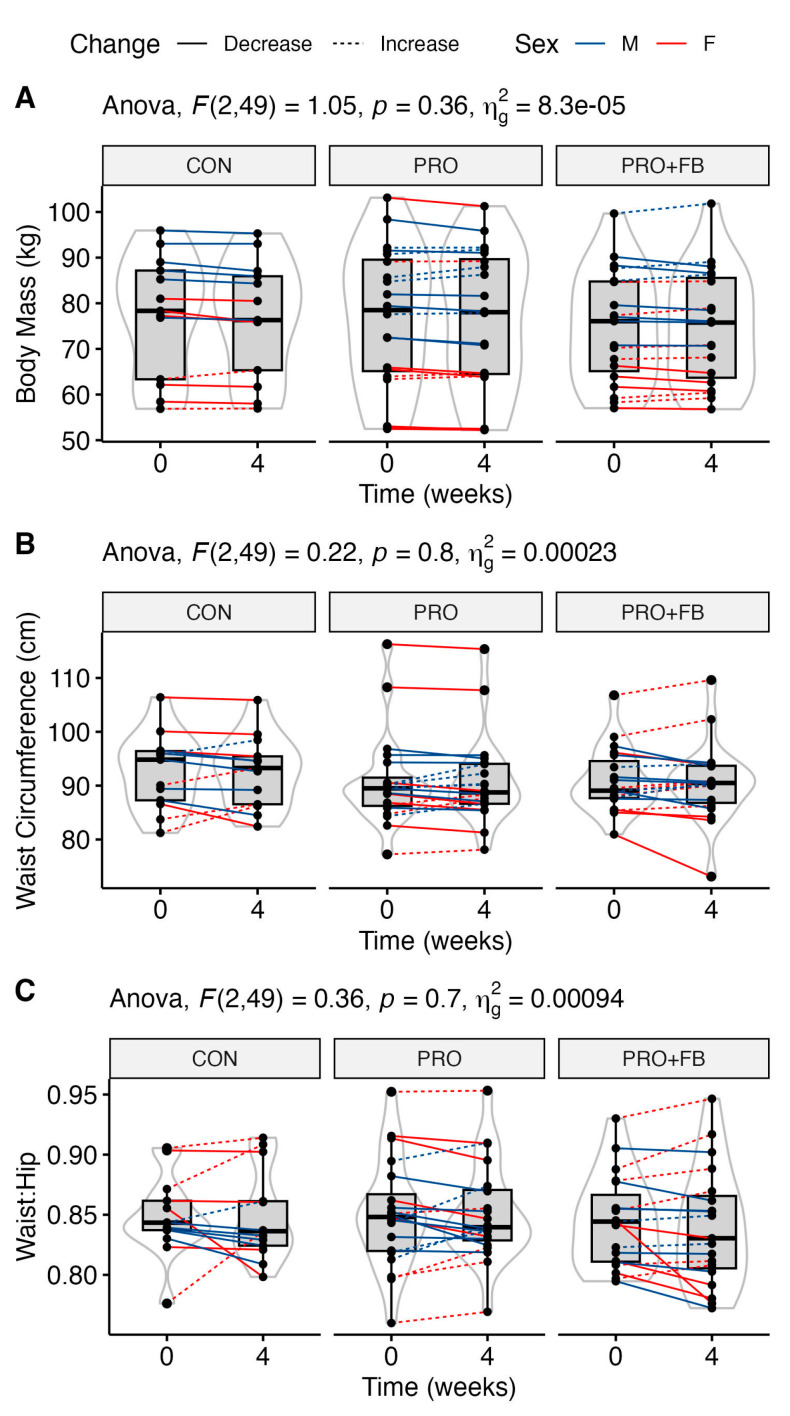
Changes in selected body mass and selected anthropometric values over the four-week intervention in each group. (**A**) Body mass in kg; (**B**) waist circumference in cm; (**C**) waist-to-hip ratio.

No significant group, time, or group × time effects were observed for body mass, waist circumference, and waist-to-hip ratio (Table 4), although a trend was present for a time main effect for body mass (*p* = 0.05). No significant differences between conditions were observed when examining change values in each condition (Table 3). Raw values and individual responses for each outcome are presented in Figure 2.

### 3.6. Metabolism and Hemodynamics

**Figure 3:**
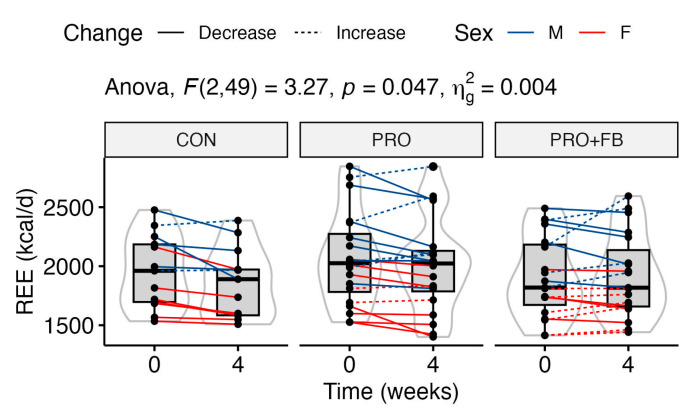
Changes in REE in kcal/day over the four-week intervention in each group.

A statistically significant group × time interaction was observed for REE values (*p* = 0.047; Figure 3). Although post hoc pairwise *t*-tests did not reveal significant differences in raw REE values between groups at either time point (*p* 0.53 to 1.0), one-way ANOVA with repeated measures indicated a significant decrease in REE in CON (−94.9 ± 107.1 kcal/d; *p* = 0.02), but not PRO (−52.3 ± 127.8 kcal/d; *p* = 0.25) or PRO + FB (+16.3 ± 132.8 kcal/d; *p* = 1.0). Additionally, a significant effect of group was observed in the one-way ANOVA test on REE change values (*p* = 0.047), with post hoc tests indicating a significant difference between PRO + FB and CON, with higher REE values in PRO + FB (111.2 kcal/d, 95% CI 2.4 to 219.9 kcal/d, *p* = 0.04). Differences between PRO and CON (42.7 kcal/d, 95% CI −65.0 to 150.3 kcal/d, *p* = 0.61) and between PRO + FB and PRO (68.5 kcal/d, 95% CI −28.3, 165.3, *p* = 0.21) were not statistically significant. 

**Figure 4:**
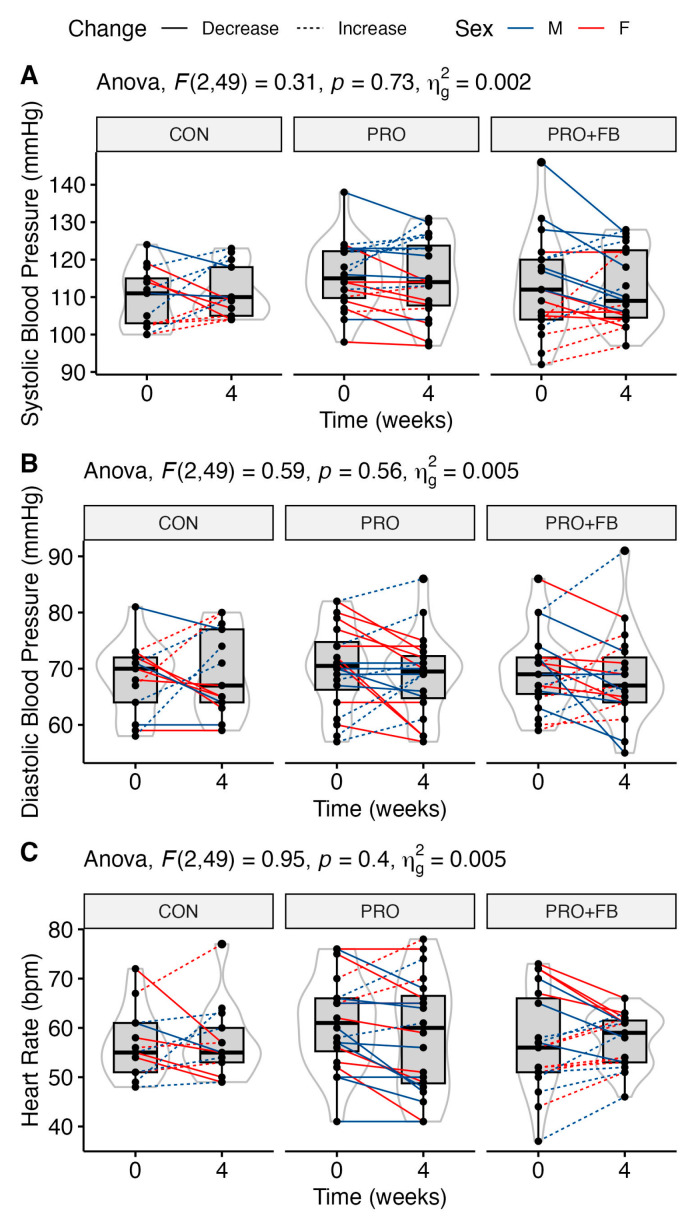
Changes in selected hemodynamic values over the four-week intervention in each group. (**A**) Systolic blood pressure in mmHg; (**B**) diastolic blood pressure in mmHg; (**C**) heart rate in beats per minute.

No significant group, time, or group × time effects were observed for RQ, heart rate, or blood pressure (Table 4). Similarly, no significant differences between conditions were observed when examining change values in each condition. Raw values and individual responses for each outcome are presented in Figure 4 and Supplementary Figure S5.

### 3.7. Sleep Quality, Eating Behavior, and Mood

At baseline, the entire sample of participants demonstrated a sleep quality score of 4.9 ± 2.2 points (range: 1–10), closely centered around the diagnostic cutoff for “poor” sleep of 5 points or greater [34]. At the second visit, participants demonstrated a mean score of 4.5 ± 2.1 (range: 0–10). No significant group, time, or group × time effects were found for this variable (Table 5). As assessed via the TFEQ-R18, participants entered the study with a mean cognitive restraint score of 13.0 ± 3.3 (range: 6–21), uncontrolled eating score of 17.7 ± 4.9 (range: 10–28), and emotional eating score of 4.8 ± 2.1 (range: 3–10). At the second visit, mean scores were 13.0 ± 4.1 (6–22), 17.4 ± 4.5 (9–28), and 4.8 ± 1.8 (3–9), respectively. No significant group, time, or group × time effects were found for any of these variables (Table 5). At baseline, the mean MFQ score across the entire sample was 6.5 ± 6.9, (range: 0–40) with scores decreasing to 5.0 ± 6.2 (range: 0–26) at the second visit. A time effect in MFQ score was present (*p* < 0.001), indicating a slight reduction in mood disturbance over time, although no significant group × time interaction was observed.

### 3.8. Side Effects

No serious adverse events were reported. From daily surveys asking “Are you experiencing any side effects you believe could be related to the dietary supplements you are consuming?”, participants in the PRO condition reported suppressed appetite (*n* = 1 participant), oily skin or acne (*n* = 1), difficulty consuming placebo supplement (*n* = 2), increased energy (*n* = 2), stomach discomfort (*n* = 1), motion sickness (*n* = 1), headache (*n* = 1), gas (*n* = 1), and increased hunger (*n* = 1). In the PRO + FB condition, reports included gas (*n* = 1 participant), indigestion or stomach discomfort (*n* = 3), feeling sluggish or tired (*n* = 2), headache (*n* = 1), sleep disturbances (*n* = 1), increased hunger (*n* = 1), acne (*n* = 1), and increased body temperature (*n* = 1). Side effects did not result in discontinuation in any of the groups.

### 3.9. Blinding Efficacy

At the end of the study, 9 out of 19 (47%) participants in PRO + FB were able to successfully guess that they had been provided with the active supplement (9 guessed the active supplement, 6 the placebo, and 4 answered, “I don’t know”). Overall, 13 out of 20 (65%) participants in PRO were able to successfully guess that they had been provided with the placebo (6 guessed the active supplement, 13 the placebo, and 1 answered, “I don’t know”).

## 4. Discussion

### 4.1. Body Composition, Body Mass, and Anthropometrics

In the current investigation, four weeks of daily thermogenic supplementation and whey protein ingestion (PRO + FB) did not result in appreciable changes in body mass, body composition, or other anthropometric variables when compared to no supplementation (CON) or to whey protein ingestion alone (PRO). It is important to reiterate that all participants were instructed to maintain their current eating frequency, food selection, and exercise habits throughout the four-week period, with the only specific dietary intervention being the addition of a whey protein supplement and the thermogenic or placebo supplement in the PRO + FB and PRO groups, respectively. However, it is possible that the increased protein intake observed in both PRO and PRO + FB led to increased satiety in these groups, as is commonly seen in weight loss interventions featuring high-protein diets [26,41,42]. Small changes in ad libitum eating patterns, including the observed decreases in carbohydrate intake, may have further resulted from these changes. Indeed, it has been theorized that dietary protein increases the sympathetic nervous system’s sensitivity to leptin [42], a hormone linked to satiety and spontaneous reductions in energy intake, in addition to increasing concentrations of other anorexigenic compounds such as peptide YY, glucagon-like peptide-1, and cholecystokinin [26]. 

Dietary protein has been frequently demonstrated to attenuate reductions in lean mass during weight loss across varied populations [43,44,45], including athletes [46]. Across all participants, decreases in total body mass, total fat mass, and leg fat mass demonstrated trends for statistical significance. Meanwhile, there was a possible signal for an interaction effect for changes in lean soft tissue, with values decreasing over time in CON but generally maintained in PRO and PRO + FB. It is not unreasonable to consider that the observed differences in protein intake as a result of supplementation may have improved lean mass retention to a more meaningful degree in the context of a longer intervention, though continued research is required to confirm this hypothesis. 

Previous investigations on the chronic effects of thermogenic supplementation on body composition and anthropometric variables have been equivocal. Tinsley et al. [47] reported no changes in fat mass, lean mass, or body fat percentage in either the resistance-trained males taking a commercially available thermogenic supplement or those given a placebo during a six-week structured resistance training program. Alternatively, Roberts and colleagues [11] observed a group × time effect for changes in both fat mass and body fat percentage over four weeks among a mixed-sex cohort of generally healthy college-aged individuals, with greater losses in the group taking a daily ready-to-drink thermogenic beverage compared to those given a placebo. Furthermore, correlation analyses showed caffeine intake to be weakly associated with observed changes in fat mass (*r* = 0.37; *p* = 0.04). 

It is possible that differences related to the training status of these populations may have played a role in their divergent results, as increased levels of training may make it more difficult to elicit detectable changes in body composition in response to a training, dietary, or supplementation stimulus. The fact that both the population and the results of the current investigation more closely reflect those in the study by Tinsley et al. lends credence to this theory. Indeed, Belza and colleagues [48] reported greater changes in fat mass among participants with overweight or obesity given a caffeinated thermogenic supplement during an eight-week weight loss program than those provided with a placebo. Conversely, Sowinski et al. [40] reported no differences between groups in a comparable population over 12 weeks of supplementation.

Perhaps the investigation most similar to ours in terms of design was that of Kendall and colleagues [49], which compared the use of a high-protein, energy-restricted diet with 16 g/day of whey protein, with either an active thermogenic supplement or placebo, to a non-dieting control condition in healthy females. While the authors did observe losses in body mass, fat mass, and body fat percentage at three weeks of follow-up in participants taking the supplement, these changes were no different from those seen in the participants given only the protein supplement and a placebo. In line with our results, these findings indicate that short-term use of a thermogenic supplement provided limited additional value for improving body composition in healthy young adults, although the possibility of differential effects with longer supplementation duration should be considered.

### 4.2. Resting Energy Expenditure and Hemodynamic Variables

A statistically significant group × time interaction was observed for REE, with values decreasing by approximately 95 ± 107 kcal/day in CON but more closely maintained at −52 ± 128 kcal/day in PRO and +16 ± 133 kcal/day in PRO + FB. A significant group effect was also observed in the one-way ANOVA on REE change values, with post hoc analyses demonstrating a significant difference between the change in PRO + FB as compared to CON (111 kcal/d, 95% CI 2 to 220 kcal/d, *p* = 0.04) but not between PRO and CON. Therefore, the co-ingestion of the thermogenic supplement with whey protein may exert a synergistic effect on maintaining REE during interventions similar to the present investigation.

The ingestion of similar caffeinated ready-to-drink beverages and supplements has been demonstrated to acutely increase REE in generally healthy [10,11,50] as well as resistance- and endurance-trained individuals [8,9]. Assuming proper intake of the supplement throughout the current investigation, participants in PRO + FB were estimated to be consuming approximately 348 mg caffeine per day at follow-up, whereas participants in CON and PRO were estimated to be consuming 86 and 74 mg/day, respectively. However, it should be noted that the timing of the REE measurement in the current study occurred after a minimum of eight hours of abstention from all substances, in the morning hours after a night’s sleep. Furthermore, the final dose of the thermogenic supplement was consumed in the early afternoon the day prior to the final REE assessment (~17 to 21 h before REE assessment), provided instructions were followed. As the average half-life of caffeine is five hours [51], it is unlikely that any acute effects of caffeine within the supplement would explain the demonstrated between-group differences in changes in REE over the course of the four-week intervention. 

Other substances within the supplement may also have contributed to the elevated REE in PRO + FB. For instance, P-synephrine—the active ingredient in bitter orange extract, a component of the investigative supplement—has been shown to acutely increase post-exercise energy expenditure in healthy adults, both with and without caffeine [52]. However, across the included ingredients, research examining the effects of chronic ingestion on fasting REE are sparse, limiting further inference. Additionally, the observed statistical difference between groups for change in REE may be related to differences in body composition changes that did not reach statistical significance within the constraints of the present study. For instance, conjugated linoleic acid has been demonstrated to increase fat-free mass accretion during weight regain in subjects with obesity, thus also improving REE compared to placebo [53]. Additionally, it is possible that the ingestion of the supplement’s ergogenic compounds around exercise allowed for greater training volume in subjects, specifically those engaging in resistance training. Over a period longer than the current four-week intervention, increased training volume may cumulatively result in a greater retention of lean mass during weight loss, which would be expected to result in improved maintenance of REE over time. 

Alternatively, the impact of increased protein ingestion on REE is worth considering as a possible explanation for these differences. Reductions in REE commonly occur in response to chronic energy restriction and weight loss [54]. Protein can directly promote the maintenance of REE during periods of weight loss by attenuating the loss of lean mass, a metabolically active tissue [43]. Protein also increases diet-induced thermogenesis, or the energy expended during the digestion, metabolism, and storage of nutrients, to a greater degree than fat or carbohydrate [25]. Indeed, elevated REE values have been observed for at least five hours after the consumption of a mixed meal [55], and this effect would be expected to be greater after a high-protein meal in particular. However, research examining the time course of RMR elevation after a high-energy or high-protein meal or supplement, specifically after 7 hours’ duration, is sparse [54,55]. Our ability to draw conclusions regarding the potential effect of increased protein intake on resulting REE values is therefore limited. It is additionally possible that co-supplementation with both a high-quality protein source and a thermogenic product, such as in the PRO + FB group, may enhance the observed effects of protein ingestion on REE over time. This would specifically be plausible when supplements are taken in conjunction with a structured resistance-training program, as the thermogenic product may increase lean mass accretion over the long-term through increased training performance and volume [56].

Previously, Roberts et al. [11] observed no group × time effect for change in fasted REE after 28 days of supplementation with a ready-to-drink thermogenic beverage compared to placebo. Though the population and interventions were similar to those of the current investigation, Roberts et al. did not include protein supplementation. This difference lends further credence to the potentially additive effect between the protein and thermogenic supplement in the current study, which resulted in improved REE in PRO + FB compared to CON. Interestingly, Sowinski et al. [40] reported a statistically significant increase in REE at four weeks of follow-up among generally healthy men and women given a placebo, but not those provided an insulin sensitivity-promoting agent (*Dichrostachys glomerata*) with or without caffeine. However, after 12 weeks of follow-up, this change had diminished, and statistically significant increases in both absolute and bodyweight-adjusted REE were seen only in the group taking the supplement with caffeine. However, a group × time interaction was not observed. 

Kendall et al. [49] observed no appreciable changes in hemodynamic variables such as resting heart rate or systolic and diastolic blood pressure after three weeks of supplementation with a thermogenic product in addition to whey protein, echoing our findings and those of the eight-week investigation by Belza et al. [48]. Unfortunately, fasted REE was not included as an outcome by either study, limiting further comparison with our findings.

### 4.3. Subjective Variables

No group × time effects were observed for changes in food and eating behaviors, sleep quality, or mood disturbance as assessed via our series of validated questionnaires. Across the entire sample, mood disturbance was reduced (−1.5; *p* < 0.001) over the four-week intervention as assessed via the MFQ. As all participants were enrolled between the months of January and April and completed the study between February and May, a small seasonal effect related to increased daylight is possible. To the authors’ knowledge, no minimal clinically important difference has been established for this scale, though scores greater than 26 out of 66 are considered indicative of depression [33]. Given the small absolute change in mean scores, it is unlikely that the observed changes are clinically meaningful.

Notably, use of the thermogenic supplement did not appear to result in decreased sleep quality over the month-long intervention as assessed via the PSQI. These results indicated that daily intake of a supplement containing 300 mg caffeine, including 150 mg taken in the afternoon hours, did not adversely affect sleep to a meaningful degree. This is a meaningful finding for potential users of thermogenic supplements containing central nervous stimulants such as caffeine, as these users would likely prefer to avoid disruptions in sleep quality and related downstream consequences on athletic performance in addition to quality of life. Changes in subjective hunger, emotional eating, and uncontrolled eating as assessed via the TFEQ-R18 were also not statistically different between groups. Therefore, use of the protein and/or thermogenic supplement did not appear to affect experiences of appetite or overeating in the current investigation, regardless of the potentially anorexigenic effects of these compounds. 

Contrary to our findings that four weeks of a caffeinated supplement (approximately 300 mg/day) did not adversely affect sleep quality in young, trained, individuals, Sowinski et al. [40] reported worsened sleep latency among generally healthy adults given a supplement containing 400 mg/day caffeine at both 4 and 12 weeks of follow-up. However, while the present investigation utilized the Pittsburgh Sleep Quality Index to assess sleep quality over the past month using a singular global score, Sowinski and colleagues elicited Likert-scale data related to four sleep-related items over the previous 48 h. The differences between these approaches and the interpretation of the resulting data make direct comparison between findings challenging. 

Sowinski and colleagues also elicited data pertaining to subjects’ appetite, hunger, satisfaction from food, fullness, energy, and overall diet quality, assessing each item on a ten-point visual analog scale. In line with our results, the authors found no differences between those given a thermogenic supplement or placebo for any outcome. However, beyond the findings of Sowinski et al., there currently exists a dearth of research related to the chronic effects of similar supplementation interventions on subjective variables in populations similar to ours. 

### 4.4. Study Strengths and Limitations

The present investigation meaningfully contributes to the current literature in several ways. It includes a mixed-sex cohort of both resistance- and endurance-trained individuals, which is reflective of the populations typically consuming thermogenic supplements [57]. Our subject pool also included only participants who were weight-stable upon enrollment and who reported consuming between 40 and 200 mg caffeine daily. This minimum required caffeine intake likely reduced the likelihood of observing extreme effects among caffeine-naïve individuals. Body composition and REE assessments were undertaken in highly standardized conditions [29,30], improving the accuracy and reliability of our findings. In addition to the participants being representative of typical thermogenic supplement consumers (i.e., generally young and recreationally active), the dietary intervention has strong generalizability to exercising individuals who intend to purchase and use dietary supplements without making other substantive changes to their dietary practices. As such, the present study may more accurately reflect real-world effects of supplementation as compared to traditional study designs that include notable dietary changes and careful oversight of participants’ dietary practices. 

One limitation of the current study is that a group consuming only a thermogenic supplement with no protein supplementation was not included. In addition, a 24 h food recall was used to elicit data related to dietary intake. Although changes in diet outside of the provided supplements was not a primary intervention of outcome of interest, it is important to note that dietary data elicited at baseline and post-intervention may not fully reflect typical intake in all participants. While participants were instructed not to change their typical dietary intake over the intervention, data related to participants’ adherence to these directions were not collected.

The limited duration of our study also deserves discussion. Though a six-week investigation [47] of resistance-trained males undergoing a structured training program during supplementation saw no differences in changes in body mass or composition, an eight-week intervention [48] among overweight and obese adults reported greater loss of fat mass in the supplement group. Meanwhile, twelve weeks of supplementation in moderately active individuals did not result in improved changes in body composition or REE when compared to placebo. Changes in fat and lean mass in response to a dietary or exercise intervention are often more gradual among individuals who already maintain a relatively lean and muscular composition. Therefore, given the physically trained nature of the participants examined within the current study, a follow-up of longer duration may have been necessary to allow such changes in body composition to become detectable. However, typical supplement users such as those reflected in our sample population may purchase thermogenic products with the intention of seeing results more rapidly, and the results of our study indicate that caution is warranted with these expectations. Furthermore, our study design allows for a direct report of the results that can be expected in this population upon purchasing and using a typical one-month supply of a thermogenic supplement.

### 4.5. Directions for Future Research

Given the relatively ad libitum nature of the intervention which required no conscious efforts to restrict energy intake or increase physical activity, it is worth considering that these gradual changes may amount to clinically meaningful effects over a longer study duration. Longer-term investigations in active and trained populations—such as those lasting 6–8 weeks or longer—are therefore needed. The understanding of the chronic effects of thermogenic supplementation would further benefit from the inclusion of outcomes similar to those in the current investigation, including fasted REE and subjective variables, such as mood, hunger, and sleep quality.

Recreationally active individuals and athletes commonly supplement with protein and/or thermogenic products such as caffeinated multi-ingredient pre-workout supplements and energy drinks [2,3,57]. Similarly to previous work by Kendall et al. [49], the findings of the current investigation highlight the need for research designed to parse the effect of commonly used co-interventions, such as concomitant use of thermogenic supplements with protein, among active individuals. Specifically, further research examining the additive influence of thermogenic products over and above those obtained by commonly used supplements such as protein and creatine is needed. 

## 5. Conclusions

To the authors’ knowledge, this is the first study to assess the chronic effects of a commercially available thermogenic supplement in combination with whey protein supplementation in active men and women. In the present investigation, four weeks of supplementation with a multi-ingredient thermogenic supplement did not result in appreciable changes in body composition, hemodynamic, or subjective variables in a population of healthy, trained young adults. However, supplementation with protein and a multi-ingredient thermogenic product maintained REE as compared to no supplementation, for which a decrease in REE was observed. This benefit for REE was observed in the context of fasted and rested assessments, with the last dose of thermogenic product consumed ~17 to 21 h before the final REE assessment due to the fact that participants were instructed to take their final dose of the supplement in the early afternoon on the day prior to their visit. As such, this change is believed to represent a chronic adaptation rather than acute response to supplementation. Despite the better maintenance of REE, no changes in heart rate or blood pressure were observed following four weeks of supplementation. The lack of observed changes in sleep quality, mood, or eating behaviors may be of further reassurance to active individuals looking to use caffeinated thermogenic supplements in conjunction with training with few undesirable changes in these variables. Further research is needed to better clarify the effects of co-supplementation with a thermogenic product versus those achieved solely with increased protein ingestion. Given the likelihood that trained populations may require a longer duration of relatively unstructured interventions to achieve detectable changes in body composition and anthropometrics, longer-term studies are needed when these are the primary outcomes. The observed benefits of combined protein and thermogenic supplementation for REE provide a rationale and a call to action for investigators to conduct longer-term studies focused on such outcomes.

## Figures and Tables

**Table 1:** Supplement facts.

	Amount per 4.5 g Serving	%Daily Value
Energy (kcal)	5	–
Total carbohydrate	1 g	<1%
Dietary fiber	0.2 g	4%
Vitamin C	174 mg	193%
Thiamin	0.56 mg	46%
Riboflavin	0.78 mg	60%
Vitamin B_3_ (niacin)	20 mg	123%
Vitamin B_6_ (pyridoxine)	0.98 mcg	58%
Vitamin B_12_ (cobalamin)	0.9 mcg	38%
Vitamin B_5_ (pantothenic acid)	1.7 mg	34%
Chromium picolinate	10 mcg	3%
Fat-Burning Matrix Acetyl L-carnitine HCl, Garcinia Cambogia fruit extract (60% hyroxycitric acid), conjugated linoleic acid (CLA), grapefruit seed extract 4:1, raspberry ketones (from raspberry fruit extract), Mangifera Indica seed extract, bitter orange fruit extract, green coffee bean extract (50% chlorogenic acid), olive leaf extract (10% oleuropein), Guggul extract powder, chromium picolinate	2003 mg	–
Immunity Booster and Prebiotic Complex L-glutamine, inulin fiber, Vitamin C (ascorbic acid)	624 mg	–
Full B-Vitamin Spectrum Niacinamide (niacin), calcium pantothenatate (pantothenic acid), pyridoxine HCl (Vitamin B_6_), riboflavin (Vitamin B_2_), thiamine mononitrate (Vitamin B_1_), cyanocobalamin (Vitamin B_12_)	24.59 mg	–

**Table 2:** Participant characteristics at baseline.

Variable	CON (*n* = 13)	PRO (*n* = 20)	PRO + FB (*n* = 19)
Sex	7 (54%) F	9 (45%) F	10 (53%) F
	6 (46%) M	11 (55%) M	9 (47%) M
Training status	7 (54%) CT	11 (55%) CT	10 (53%) CT
	5 (38%) RT	8 (40%) RT	7 (37%) RT
	1 (8%) ET	1 (5%) ET	2 (11%) ET
Height (cm)	167.8 ± 8.1	171.5 ± 10.7	170.9 ± 8.4
Body mass (kg)	77.3 ± 13.2	76.8 ± 15.4	74.8 ± 12.3
Body fat percentage	28.5 ± 7.2	26.0 ± 8.7	27.8 ± 8.0
Age (years)	23.1 ± 2.3	23.9 ± 3.3	23.5 ± 3.0
Caffeine intake (mg per day)	164 ± 48	142 ± 54	134 ± 54
Training age (years)	8.4 ± 4.8	7.2 ± 4.7	8.5 ± 5.3
Training frequency (days per week)	4.7 ± 1.1	4.7 ± 1.3	4.3 ± 1.4
Physical activity (MET-min per week)	4251 ± 2552	4506 ± 2889	3701 ± 1990
Physical activity energy expenditure (kcal per day)	828 ± 518	919 ± 745	678 ± 350
Energy intake (kcal per day)	2008 ± 760	2229 ± 947	2276 ± 881
Protein intake (g per day)	112 ± 64	131 ± 72	115 ± 52
Fat intake (g per day)	81 ± 43	91 ± 53	95 ± 46
Carbohydrate intake (g per day)	207 ± 58	225 ± 90	243 ± 97
MFQ score	6.2 ± 4.1	6.5 ± 5.6	6.8 ± 9.5
PSQI score	5.1 ± 1.7	4.7 ± 2.2	5.0 ± 2.6
TFEQ cognitive restraint score	13.5 ± 2.2	12.1 ± 3.7	13.5 ± 3.6
TFEQ uncontrolled eating score	18.1 ± 5.4	16.7 ± 6.2	17.4 ± 4.5
TFEQ emotional eating score	5.6 ± 2.5	4.7 ± 1.6	4.5 ± 2.1

MFQ: Mood and Feelings Questionnaire; PSQI: Pittsburgh Sleep Quality Index; TFEQ: Three-Factor Eating Questionnaire.

**Table 3:** Results for dietary and energy expenditure outcome variables.

	CON Change		PRO Change		PRO + FB Change		One-Way ANOVA		Two-Way ANOVA		
Variable	Mean	SD	Mean	SD	Mean	SD	*p* (Group)	GES	*p* (Group)	*p* (Time)	*p* (Group × Time)
Energy intake (kcal/day)	−128	360	−276	727	−154	556	0.72	0.01	0.67 ^#^	0.17 ^#^	0.76 ^#^
Protein intake (g/day)	−16	26	13	59	19	36	0.03 *^#^	0.11 ^#^	0.24	0.40	0.08
Fat intake (g/day)	−1	35	−15	39	−10	37	0.60	0.02	0.76	0.11	0.60
Carbohydrate intake (g/day)	−8	38	−52	69	−36	62	0.13	0.08	0.58	<0.001 *	0.13
Caffeine intake (mg/day)	−78	44	−68	64	214	64	<0.0001 *	0.85	0.03	<0.0001 *	<0.0001 *
PA (MET-min/week)	−4	2307	−226	2278	−443	1247	0.82	0.01	0.32	0.42	0.82
PAEE (kcal/day)	−6	453	−93	547	−73	210	0.82	0.01	0.25	0.35	0.84

One-way ANOVA was performed using change values, and two-way ANOVA was performed using raw values. ^#^ result based on rank-based test due to assumption violation and different results between parametric and rank-based tests. Effect size accompanying the Kruskal–Wallis test is the eta-squared effect size. * statistically significant at *p* < 0.05. ANOVA: analysis of variance; GES: generalized eta-squared; PA: physical activity; PAEE: physical activity energy expenditure.

**Table 4:** Results for body composition, anthropometric, metabolic and hemodynamic outcome variables.

	CON Change		PRO Change		PRO + FB Change		One-Way ANOVA		Two-Way ANOVA		
Variable	Mean	SD	Mean	SD	Mean	SD	*p* (Group)	GES	*p* (Group)	*p* (Time)	*p* (Group × Time)
Body mass	−0.65	1.07	−0.36	1.23	−0.03	1.27	0.36	0.04	0.88	0.05	0.36
BFP (total)	0.12	1.38	−0.56	1.01	−0.20	0.89	0.21	0.06	0.59	0.16	0.21
BFP (arms)	−0.09	1.22	−0.42	1.18	−0.14	1.11	0.66	0.02	0.53	0.19	0.66
BFP (legs)	0.14	1.22	−0.28	0.99	−0.36	0.70	0.33	0.04	0.53	0.23	0.33
BFP (trunk)	0.20	1.95	−0.87	1.34	−0.11	1.41	0.12	0.08	0.72	0.24	0.12
FM (total)	−0.13	1.17	−0.48	0.84	−0.14	0.94	0.47	0.03	0.72	0.08	0.47
FM (arms)	0.00	0.18	−0.06	0.12	−0.01	0.14	0.41	0.04	0.60	0.24	0.41
FM (legs)	−0.07	0.38	−0.10	0.32	−0.09	0.29	0.96	0.00	0.59	0.06	0.96
FM (trunk)	−0.06	0.77	−0.31	0.53	−0.04	0.62	0.37	0.04	0.86	0.13	0.37
VAT mass	−15.00	58.48	−22.95	77.26	7.84	59.48	0.34	0.04	0.92	0.29	0.34
LST (total)	−0.58	1.09	0.15	1.04	0.05	0.81	0.10	0.09	0.78	0.36	0.10
LST (arms)	0.00	0.20	−0.03	0.21	0.00	0.20	0.86	0.01	0.70	0.77	0.86
LST (legs)	−0.27	0.45	0.00	0.40	−0.01	0.38	0.14	0.08	0.78	0.12	0.14
LST (trunk)	−0.31	0.73	0.21	0.75	0.07	0.62	0.13	0.08	0.80	0.93	0.13
SCF	0.01	0.48	−0.09	0.29	−0.01	0.25	0.63	0.02	0.68	0.52	0.63
WC	−0.06	2.71	0.14	1.71	−0.36	2.73	0.80	0.01	0.76	0.78	0.80
WHR	0.00	0.03	0.00	0.02	0.00	0.02	0.70	0.01	0.79	0.77	0.70
REE	−94.92	107.10	−52.25	127.81	16.26	132.84	0.047 *	0.12	0.47	0.02 *	0.047 *
RQ	0.00	0.06	0.00	0.06	−0.01	0.08	0.84	0.01	0.17	0.61	0.84
HR	0.39	7.57	−2.30	5.91	0.21	6.58	0.40	0.04	0.54	0.55	0.40
SBP	1.54	8.36	−0.45	5.94	−0.42	9.04	0.73	0.01	0.33	0.84	0.73
DBP	1.23	8.13	−1.40	6.63	−0.95	6.74	0.56	0.02	0.94	0.71	0.56

One-way ANOVA was performed using change values, and two-way ANOVA was performed using raw values. * statistically significant at *p* < 0.05. BFP: body fat percentage (%); DBP: diastolic blood pressure (mmHg); FM: fat mass (kg); HR: heart rate (beats per min); LST: lean soft tissue (kg); REE: resting energy expenditure (kcal/day); RQ: respiratory quotient; SBP: systolic blood pressure (mmHg); SCF: subcutaneous fat thickness (cm); VAT: visceral adipose tissue (g); WC: waist circumference (cm); WHR: waist-to-hip ratio.

**Table 5:** Results for sleep quality, eating behavior, and mood variables.

	CON Change		PRO Change		PRO + FB Change		Kruskal–Wallis Test		Two-Way ANOVA on Ranks		
Variable	Mean	SD	Mean	SD	Mean	SD	*p* (Group)	ES (H)	*p* (Group)	*p* (Time)	*p* (Group × Time)
Sleep quality (PSQI)	−0.6	1.5	−0.7	1.8	0.1	2.0	0.40	0.02	0.53	0.10	0.34
Cognitive restraint (TFEQ)	−0.5	1.8	0.6	2.7	−0.3	2.7	0.38	0.00	0.60	0.55	0.30
Uncontrolled eating (TFEQ)	0.2	2.6	−0.5	2.9	−0.4	3.2	0.86	0.03	0.87	0.86	0.68
Emotional eating (TFEQ)	−0.2	1.4	−0.1	1.1	0.1	2.3	0.43	0.01	0.34	0.82	0.72
Mood disturbance (MFQ)	−0.7	4.1	−2.2	4.7	−1.4	5.7	0.69	0.03	0.79	<0.001 *	0.32

ES: eta-squared; H: H-statistic; MFQ: Mood and Feelings Questionnaire; PSQI: Pittsburgh Sleep Quality Index; TFEQ: Three-Factor Eating Questionnaire. * statistically significant at *p* < 0.001.

## Data Availability

Data may be available from the corresponding author upon reasonable request and pending relevant approvals.

## Supplementary Materials

The following supporting information can be downloaded at: https://www.mdpi.com/article/10.3390/nu15224806/s1, Figure S1: Changes in segmental body fat percentage in each group; Figure S2: Changes in segmental and visceral fat mass in each group; Figure S3: Changes in segmental lean soft tissue in each group; Figure S4: Changes in subcutaneous fat thickness in each group; Figure S5: Changes in respiratory quotient in each group; Table S1: Baseline characteristics stratified by sex and group; Table S2: Three-way ANOVA results with sex as a factor.
