# Supplementary figures and images for: Chronic Thermogenic Dietary Supplement Consumption: Effects on Body Composition, Anthropometrics, and Metabolism
Source: Nutrients. 2023 Nov 17;15(22):4806. doi: 10.3390/nu15224806 (PMC10674526; doi:10.3390/nu15224806)

Change — Decrease ..... Increase Sex — M — F

**A** Anova,  $F(2,49) = 0.42$ ,  $p = 0.66$ ,  $\eta_g^2 = 6e-05$

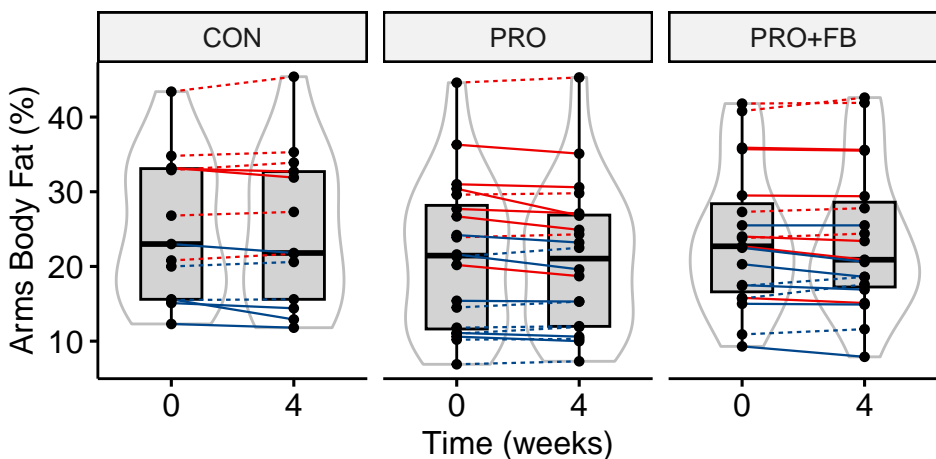

**B** Anova,  $F(2,49) = 1.14$ ,  $p = 0.33$ ,  $\eta_g^2 = 0.00014$

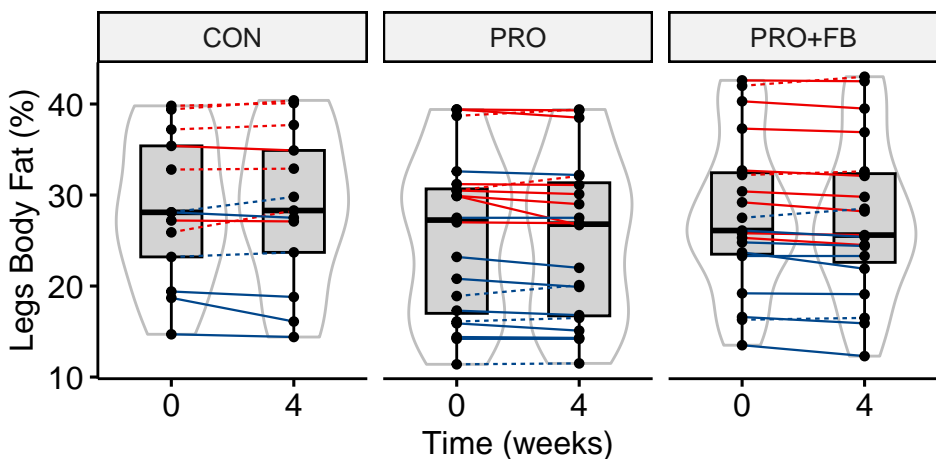

**C** Anova,  $F(2,49) = 2.19$ ,  $p = 0.12$ ,  $\eta_g^2 = 0.00073$

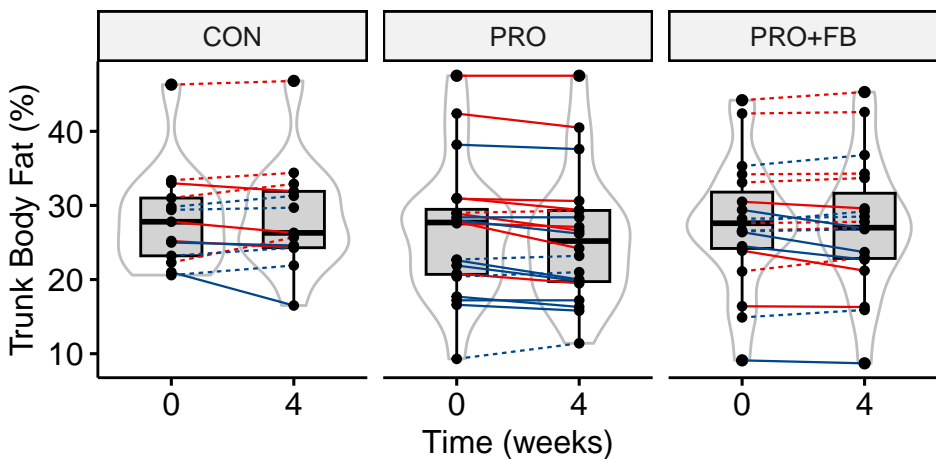

Supplement: Supplementary file 1 [file nutrients-15-04806-s001.zip › Figure S1.pdf]

Change — Decrease ..... Increase Sex — M — F

**A** Anova,  $F(2,49) = 0.91$ ,  $p = 0.41$ ,  $\eta_g^2 = 0.00026$

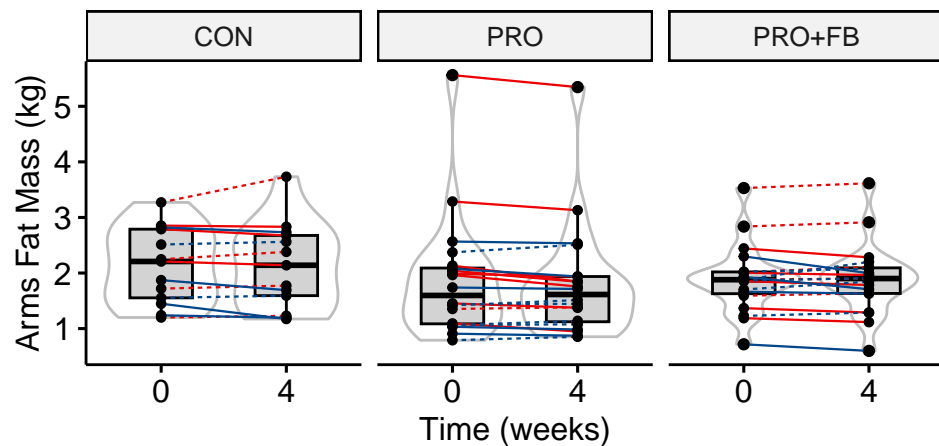

**B** Anova,  $F(2,49) = 0.04$ ,  $p = 0.96$ ,  $\eta_g^2 = 6.1e-06$

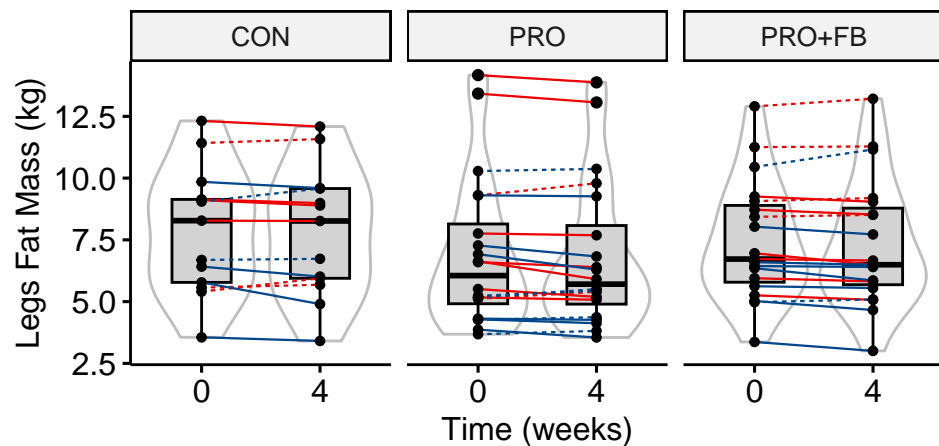

**C** Anova,  $F(2,49) = 1.01$ ,  $p = 0.37$ ,  $\eta_g^2 = 0.00026$

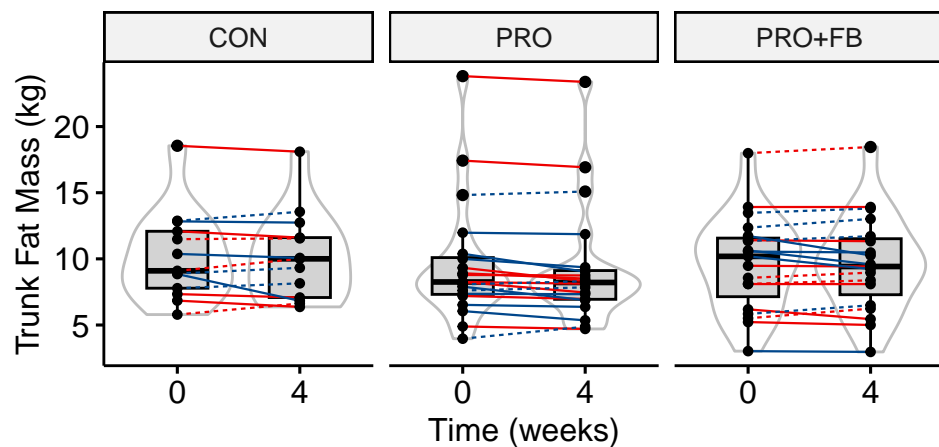

**D** Anova,  $F(2,49) = 1.09$ ,  $p = 0.34$ ,  $\eta_g^2 = 0.00081$

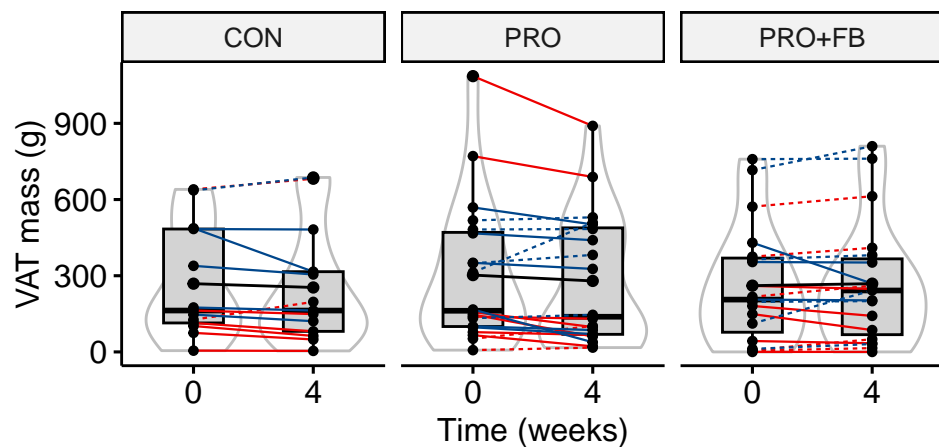

Supplement: Supplementary file 1 [file nutrients-15-04806-s001.zip › Figure S2.pdf]

Change — Decrease ..... Increase Sex — M — F

**A** Anova,  $F(2,49) = 0.15$ ,  $p = 0.86$ ,  $\eta_g^2 = 1.4e-05$

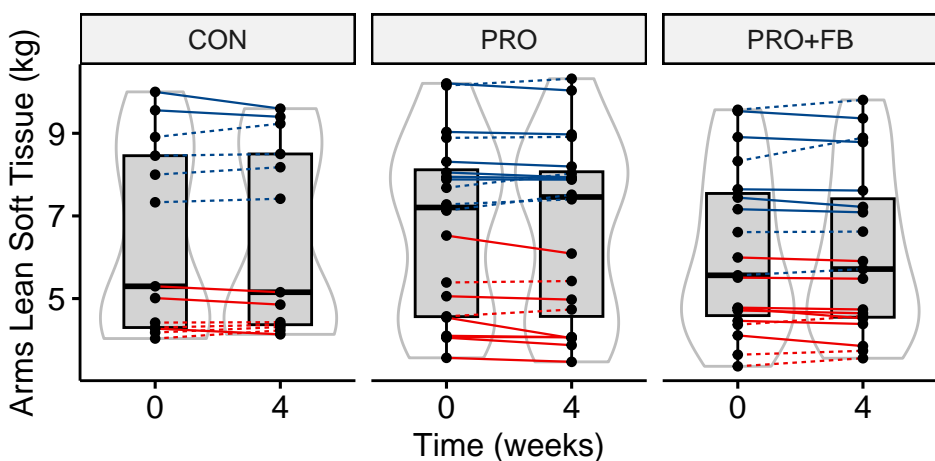

**B** Anova,  $F(2,49) = 2.08$ ,  $p = 0.14$ ,  $\eta_g^2 = 0.00018$

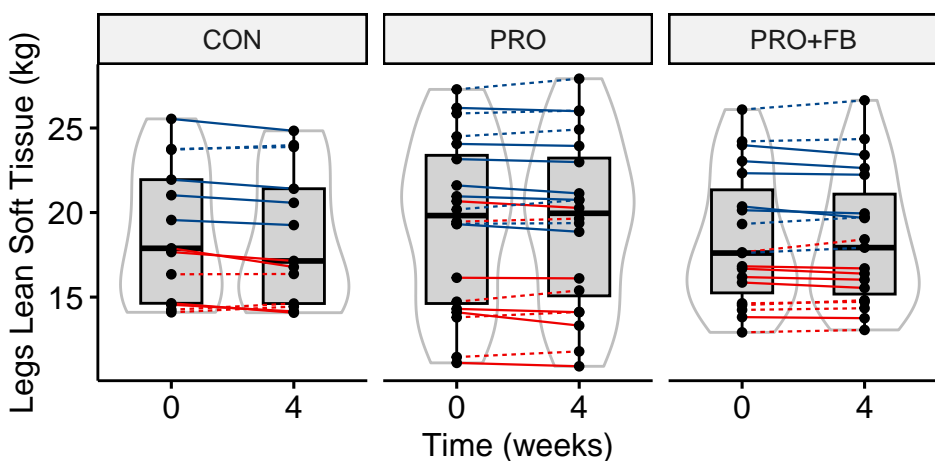

**C** Anova,  $F(2,49) = 2.16$ ,  $p = 0.13$ ,  $\eta_g^2 = 0.00036$

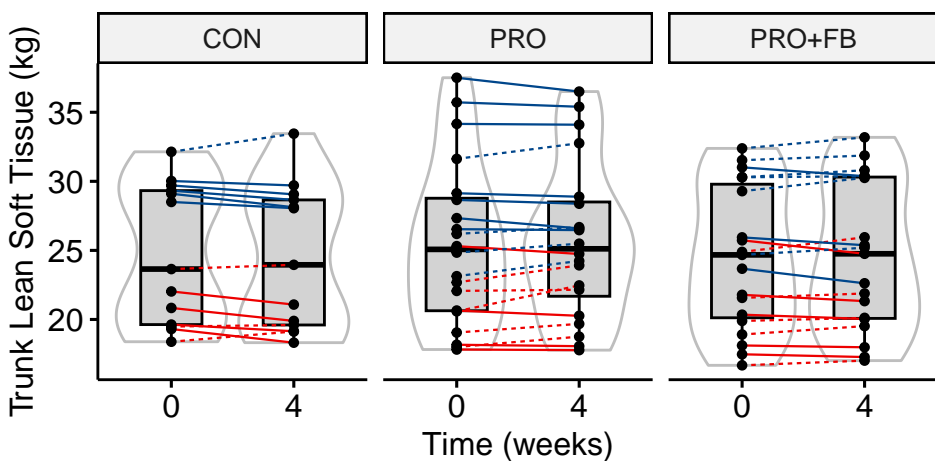

Supplement: Supplementary file 1 [file nutrients-15-04806-s001.zip › Figure S3.pdf]

Change — Decrease ..... Increase      Sex — M — F

Anova,  $F(2,49) = 0.47$ ,  $p = 0.63$ ,  $\eta_g^2 = 0.00086$

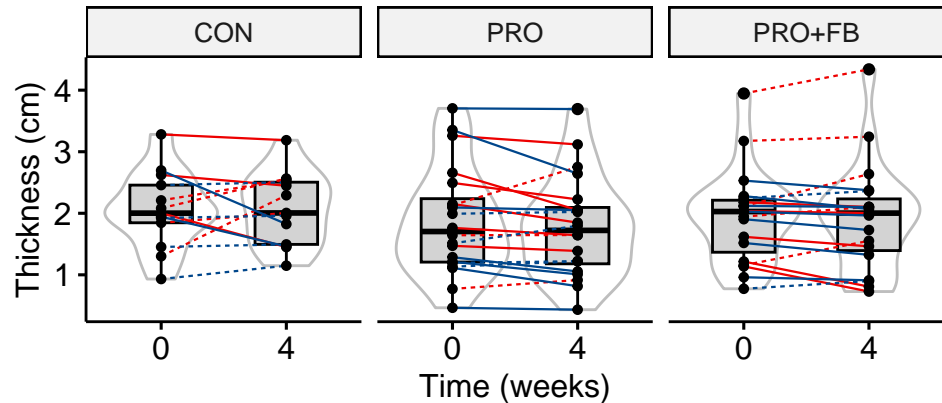

Supplement: Supplementary file 1 [file nutrients-15-04806-s001.zip › Figure S4.pdf]

Change — Decrease ..... Increase      Sex — M — F

Anova,  $F(2,49) = 0.001$ ,  $p = 1$ ,  $\eta_g^2 = 1.3e-05$

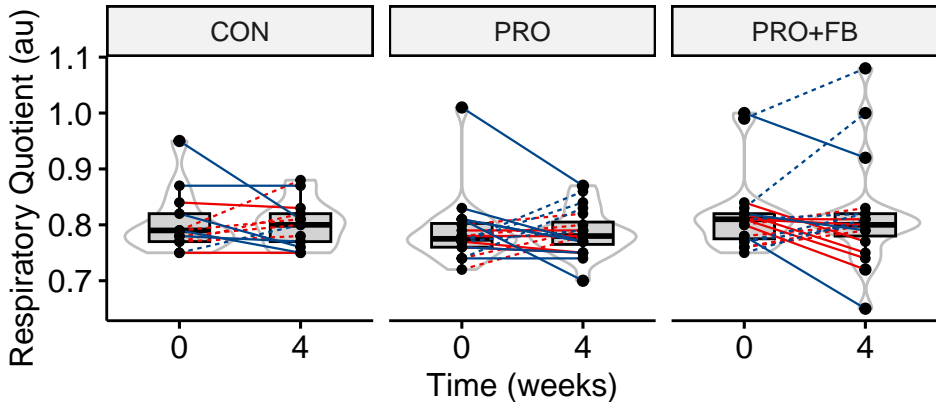

Supplement: Supplementary file 1 [file nutrients-15-04806-s001.zip › Figure S5.pdf]
